# Genomic landscape of metastatic breast cancer (MBC) patients with methylthioadenosine phosphorylase (*MTAP*) loss

**DOI:** 10.18632/oncotarget.28376

**Published:** 2023-03-11

**Authors:** Maroun Bou Zerdan, Prashanth Ashok Kumar, Elio Haroun, Nimisha Srivastava, Jeffrey Ross, Abirami Sivapiragasam

**Affiliations:** ^1^Department of Internal Medicine, SUNY Upstate Medical University, Syracuse, NY 13210, USA; ^2^Department of Internal Medicine, Division of Hematology Oncology, SUNY Upstate Medical University, Syracuse, NY 13210, USA; ^3^SUNY Upstate Medical University, Syracuse, NY 13210, USA; ^4^Foundation Medicine, Inc., Morrisville, NC 27560, USA; ^5^Departments of Pathology and Urology, SUNY Upstate Medical University, Syracuse, NY 13210, USA

**Keywords:** breast cancer, metastatic, MTAP loss

## Abstract

Introduction: Homozygous deletion of *MTAP* upregulates *de novo* synthesis of purine (DNSP) and increases the proliferation of neoplastic cells. This increases the sensitivity of breast cancer cells to DNSP inhibitors such as methotrexate, L-alanosine and pemetrexed.

Materials and Methods: 7,301 cases of MBC underwent hybrid-capture based comprehensive genomic profiling (CGP). Tumor mutational burden (TMB) was determined on up to 1.1 Mb of sequenced DNA and microsatellite instability (MSI) was determined on 114 loci. Tumor cell PD-L1 expression was determined by IHC (Dako 22C3).

Results: 208 (2.84%) of MBC featured *MTAP* loss. *MTAP* loss patients were younger (*p* = 0.002) and were more frequently ER− (30% vs. 50%; *p* < 0.0001), triple negative (TNBC) (47% vs. 27%; *p* < 0.0001) and less frequently HER2+ (2% vs. 8%; *p* = 0.0001) than *MTAP* intact MBC. Lobular histology and *CDH1* mutations were more frequent in *MTAP* intact (14%) than *MTAP* loss MBC (*p* < 0.0001). *CDKN2A* (100%) and *CDKN2B* (97%) loss (9p21 co-deletion) were significantly associated with *MTAP* loss (*p* < 0.0001). Likely associated with the increased TNBC cases, BRCA1 mutation was also more frequent in *MTAP* loss MBC (10% vs. 4%; *p* < 0.0001). As for immune checkpoint inhibitors biomarkers, higher TMB >20 mut/Mb levels in the *MTAP* intact MBC (*p* < 0.0001) and higher PD-L1 low expression (1–49% TPS) in the *MTAP* loss *MTAP* (*p* = 0.002) were observed.

Conclusions: *MTAP* loss in MBC has distinct clinical features with genomic alterations (GA) affecting both targeted and immunotherapies. Further efforts are necessary to identify alternative means of targeting PRMT5 and MTA2 in *MTAP*-ve cancers to benefit from the high-MTA environment of *MTAP*-deficient cancers.

## INTRODUCTION

Breast cancer is the most diagnosed malignancy worldwide and a leading cause of cancer-related death in women [1]. Recent advancements in diagnostic and therapeutic modalities have led to improved survival and prognosis. Tumor metastasis is one of the driving factors for treatment failure and mortality from cancer with underlying molecular mechanism still poorly understood [2, 3]. One of such alterations includes loss of tumor suppressor gene [4]. Treatment modalities aimed at halting or possibly reversing the molecular pathway leading to metastasis hold promise for effectively treating cancers.

Breast cancers can be broadly divided as per their hormone receptor (HR) and human epidermal growth factor receptor 2 (HER2) status into HR positive, HER2 positive and triple negative breast cancer (TNBC) [5]. In addition to surgery, radiation therapy, endocrine therapy and hormone therapy, tumor-tailored treatment can be provided with therapies targeting HER2, PIK3CA, TRK, CDK4/6, BRCA1/2, and VEGF and PDL1 receptors. Research is underway for multiple other promising therapies [6, 7].

**Figure 1:**
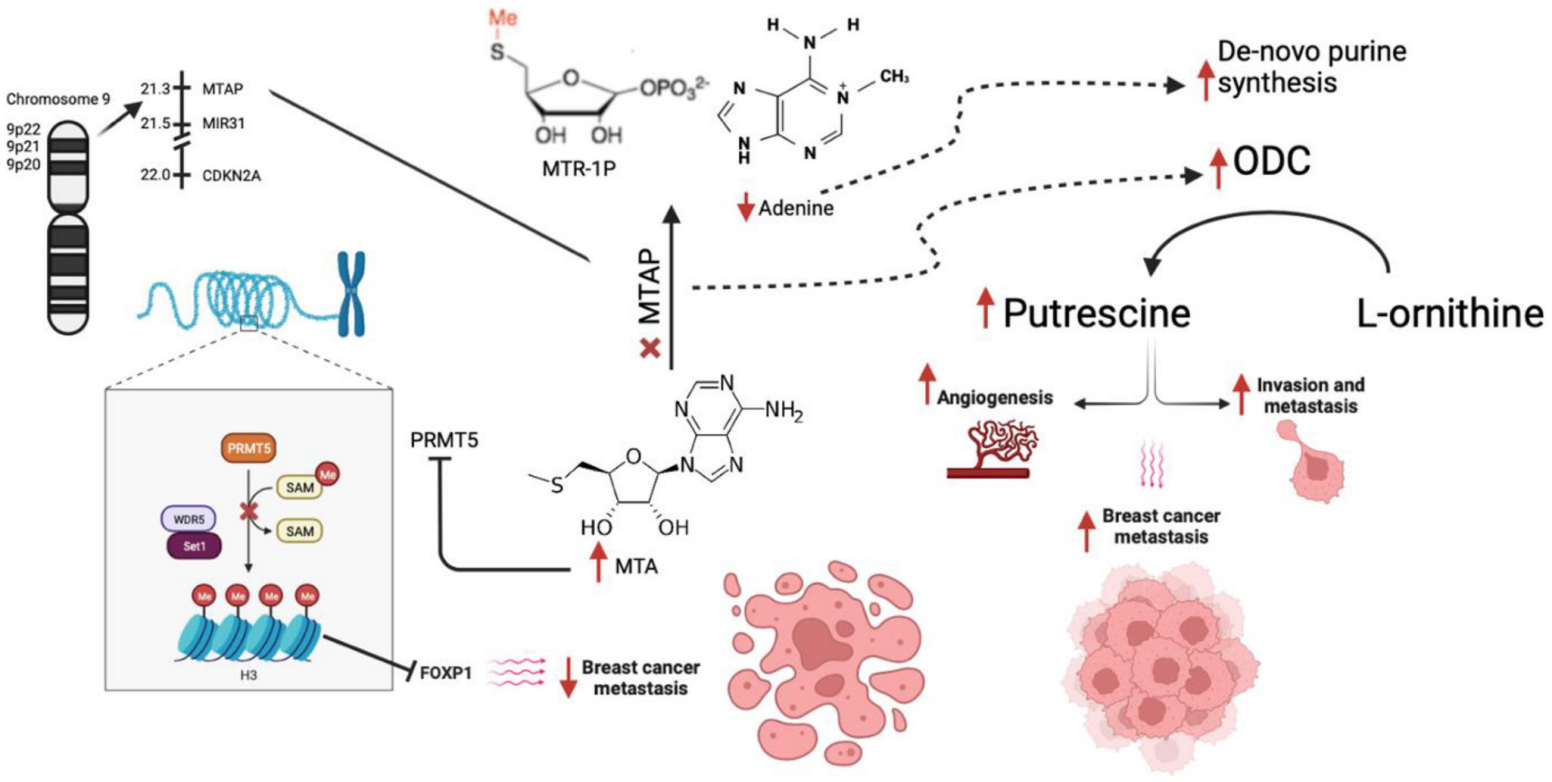
Mechanism of action of MTAP along with downstream effects from its loss. Abbreviations: MTAP: methylthioadenosine phosphorylase; MTR1P: 5-methylthioribose-1-phosphate; ODC: Ornithine decarboxylase; PRMT5: Protein Arginine Methyltransferase 5.

5′Methylthioadenosine phosphorylase (*MTAP*) is a key enzyme in the polyamine pathway and aids in catabolism of 5′Deoxy-5′-Methythioadenosine (MTA) leading to formation of methionine and adenine. *MTAP* gene is located at 9P21 surrounded by miR-31 and CDK2NA and has been reported to serve as a tumor suppressor gene [8–10]. *MTAP* deletion leads to low levels of adenine leading to cellular dependence on *de novo* purine synthesis and accumulation of MTA which in turn inhibits PRMT5 [11]. Most tumor cells have *MTAP*, P16 and other tumor suppressor genes located on 9P21 such as CDKN2A and CDKN2B making it a poor target for therapeutic regimens [11]. MTA accumulation in *MTAP* deleted cells creates a hypomorphic PRMT5 state that is sensitized towards further PRMT5 inhibition making PRMT5 inhibitors a potential therapy for *MTAP* deleted cancers [8]. PRMT5 inhibition leads to reduced histone methylation of which eventually leads to decrease *FOXP1* expression (Figure 1). This not only creates sensitivity to PRMT5 targeting, but also leads to cell apoptosis and decreased metastasis [12, 13]. *MTAP* downregulation also promotes tumor metastasis by activating the GSK3B/slug/E-cadherin axis in esophageal squamous cell carcinoma [14]. In breast cancer, *MTAP* downregulation activates ornithine decarboxylase (ODC) which in turn leads to formation of putrescine which promotes tumor migration, invasion and angiogenesis [15]. Cytotoxicity assays with inhibitors of *de novo* adenine synthesis, 5-fluorouracil (5-FU), methotrexate (MTX) and 5′aza-deoxycytidine (AZA) after *MTAP* gene knockdown in breast cancer cell lines have shown an increased sensitivity to 5-FU [4].

## RESULTS

Overall, 7301 cases of metastatic breast cancer underwent hybrid capture based comprehensive genomic profiling (CGP). 208 patients out of 7301 (2.84%) were noted to have *MTAP* loss (Table 1). The median age of patients with *MTAP* loss was 54.5 years compared to 57.8 years in *MTAP* intact (*P* = 0.002). Tumors with *MTAP* loss were noted to have lesser ER expression (50%) compared to *MTAP* intact (70%) (*p* < 0.001). Similarly, HER2 expression was less frequently noted in *MTAP* loss tumors as well (1.92% vs 7.8% in *MTAP* intact, *p* < 0.05). Triple negative status was noted more frequently noted in *MTAP* loss (47.28%) than *MTAP* intact (27%) (*p* < 0.05).

**Table 1:** Targetable and non-targetable GA along with number of cases in our cohort and their characteristics

		Cases with *MTAP* Intact	Cases with *MTAP* Loss
Number of Cases		7093	208
Mean Age^*^		57.8	54.5
ER+/PR+Status by IHC^**^		70.0%/49.0%	50.00%/29.90%
HER2+Amplification by CGP^*^		7.80%	1.92%
TNBC Status^*^		27.00%	47.28%
Driver Alterations/sample^**^		5.7	8.81
**Non-targetable GA (%)**			
TP53^*^		51.70	61.30
CDKN2A^**^		3.10	100.00
CDKN2B^**^		1.30	96.70
RB1^*^		7.20	1.40
CDH1^*^		14.30	0.90
**Targetable GA (%)**			
PTEN^*^		13.10	21.70
PIK3CA^**^		36.8	23.60
*NF1*		6.40	9.90
BRCA1^**^		3.70	9.90
*ERBB2* amplification^*^		7.80	1.92
*ERBB2* sequence mutation^*^		11.20	6.60
EGFR^*^		2.60	5.20
**Immuno-Oncology Drug Biomarkers**			
MSI High	Frequency	0.03%	0.05%
	Cases Tested	7077	205
*CD274* (*PD-L1*) Amp		1.10%	2.80%
*STK11* Inactivating GA		1.50%	4.20%
Median TMB		2.5	2.5
TMB >10%/>20%		7.84%/7.40%	5.32%/0.96%
PD-L1 Positive IC Expression (Dako 22C3)	Low (1–49%)^*^	11.45%	42.90%
	High (> 50%)	2.86%	0.00%

^*^Significant (*P* < 0.05), ^**^(*P* < 0.0001).

Among currently non-targetable mutations, *MTAP* loss tumors had higher frequency of TP53 (61.30 % vs. 51.70, *p* < 0.05), CDKN2A (100% vs. 3.10%, *p* < 0.001) and CDKN2B (96.70% vs. 1.30%, *p* < 0.001) *MTAP* intact tumors had higher frequency of RB1 (7.20% vs. 1.40%, *p* < 0.05) and CDH1 (14.30% vs. 0.90%, *p* < 0.05).

Among targetable mutations, *MTAP* loss tumors had higher frequency of PTEN (21.70% vs. 13.10%, *p* < 0.05), BRCA1 (9.90% vs. 3.70%, *p* < 0.001) and EGFR mutation (5.20% vs. 2.60%, *p* < 0.05). *MTAP* intact tumors had higher frequency of PIK3CA (36.8% vs. 23.60%, *p* < 0.001), ERBB2 amplification (7.80% vs. 1.92%, *p* < 0.05) and ERBB2 sequence mutation (11.20% vs. 6.60%, *p* < 0.05). The expression of NF1 was not statistically significant with 9.90% in *MTAP* loss vs. 6.40 in *MTAP* intact.

**Figure 2:**
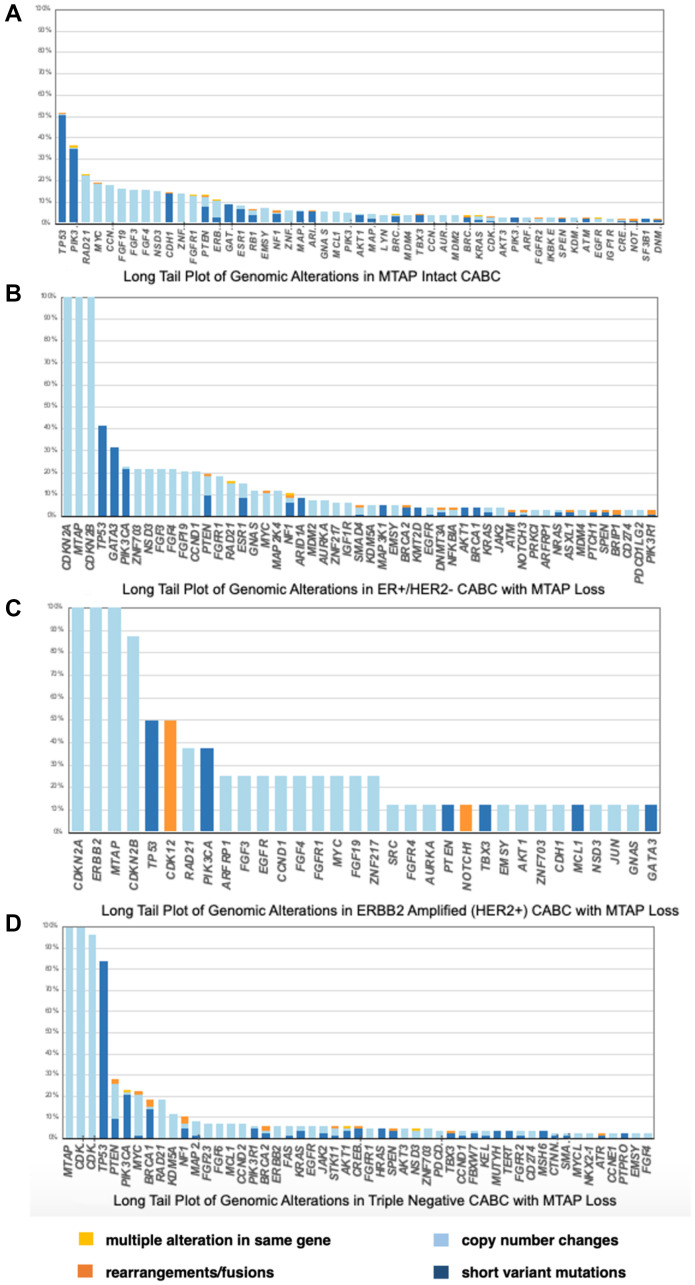
(**A**) Long Tail Plot of Genomic Alterations in MTAP Intact clinically advanced breast cancer. (**B**) Long Tail Plot of Genomic Alterations in ER+/HER2− clinically advanced breast cancer with MTAP Loss. (**C**) Long Tail Plot of Genomic Alterations in ERBB2 Amplified (HER2+) clinically advanced breast cancer with MTAP Loss. (**D**) Long Tail Plot of Genomic Alterations in Triple Negative clinically advanced breast cancer with MTAP Loss.

The tumors were also tested for checkpoint inhibitor biomarkers. The analysis included 205 out of 208 *MTAP* loss and 7077 out of 7093 *MTAP* intact tumors. The PDL-1 low status analyzed by Dako 22C3 was statistically higher in *MTAP* loss compared to *MTAP* intact tumors (42.90% vs. 11.45%, *p* < 0.05). PDL-1 high status (>50%) was not noted in the *MTAP* loss tumors and 2.86% in *MTAP* intact, however this finding was not statistically significant. These results can be seen in Figure 2.

## DISCUSSION

*MTAP* is an important enzyme found in almost all tissues in the body. It is an important enzyme in the methionine salvage pathway, responsible for regenerating methionine and adenine, which is in turn essential for the cell cycle [13]. Following the concepts of synthetic lethality, when *MTAP* is lost in a tumor cell, MTA will build up inside the cell leading to more suppression of PRMT5, thereby increasing their vulnerability to inhibition [16, 17]. PRMT5 inhibitor molecules like GSK332659513, PRT81114 and JNJ-6461917815 are currently under investigation in advanced solid malignancies including breast cancer. Safety data on GSK3326595, were recently reported by the phase 1 METEOR-1 trial (NCT02783300) which included breast cancer patients. Phase 2 portion of the study is currently underway [18]. PRMT5 is an inhibitor of tumor suppressor genes and thus enables the unchecked proliferation of cancer cells. PRMT5 induces methylation of p53 and disrupts its ability to cause death of malignant cells. It also promotes cyclin kinase-dependent neoplastic growth. The clinical paradigm of *MTAP* deficient cells, by building up MTA, which is a potent inhibitor of PRTM5, was studied as early as 1981 [18]. Another evolving target of interest are the methionine adenosyltransferases MAT1a and MAT2a. They are important cofactors in the polyamine biosynthesis cycle and play an essential role in the growth and survival of cells. *In vivo* models have shown that MAT2a knockdown reduced the growth and development of *MTAP* deficient tumor cells [19]. IDE397, a small molecule inhibitor of MAT2A, is under investigation as a part of a Phase 1 trial for advanced solid tumors with *MTAP* deletion [20].

In our cohort of MBC, the frequency of *MTAP* loss was 2.84% (208/7301). In the COSMIC gene database, *MTAP* copy number variation loss was reported in 2.55% (38/1492) breast cancer samples [21]. In the AACR GENIE portal, *MTAP* deletion was reported in 2.9% (138/15210) breast cancer specimens [4, 22]. These findings are consistent with our analysis. Although literature on *MTAP* loss in breast cancer is scarce, *MTAP* and CDKN2A loss co-occur with concordance in up to 90% of the cases, enabling indirect estimation of *MTAP* loss. A study evaluating this, estimated *MTAP* loss at around 16% (19/119) from frozen section specimens. Larger datasets as mentioned above revealed a lower number, suggesting that the small size could be the limitation of this study [4].

We provide one of the first large analyses of the spectrum of GA occurring in *MTAP* deleted MBC with the hope that this would enable identifying potential therapeutic agents in the future.

Even though breast cancer is becoming more common worldwide, its prognosis has improved thanks to advances in early detection and treatment. Currently, distant metastasis has the largest influence on a breast cancer patient’s prognosis. The five-year survival rate of breast cancer patients without metastasis is over 80% [23], but that of patients with metastasis is only around 25% [2, 3]. Yet, the molecular basis for the spread of breast cancer remains poorly understood.

In many human malignancies, such as leukemia [24], lymphoma [25], lung cancer [26], pancreatic cancer [27], and melanoma [28, 29], *MTAP* is frequently suppressed or absent, making it a potential target for cancer treatment. However, uncertainty still exists regarding *MTAP*’s clinical and biological impact on breast cancer metastasis.

Similarly, Zhang et al. revealed for the first time a positive correlation between reduced *MTAP* expression and tumor recurrence in breast cancer patients, indicating that *MTAP* may be crucial to the malignant development of breast cancer [15]. This study showed that, in an orthotopic breast cancer model using BT20 cells, *MTAP* downregulation could greatly accelerate both tumor development and metastasis. *MTAP* expression also differs by breast cancer type: studies have shown that TNBC cells express significantly less *MTAP* than the more differentiated group made up of Luminal-A breast tumors, this would open the door for novel therapeutical strategies for the treatment of TNBC where endocrine or targeted therapy are usually ineffective [30].

The results of cytotoxicity assays using inhibitors of *de novo* adenine synthesis (5-FU, AZA, and MTX) after MTAP gene knockdown showed an increased sensitivity, primarily to 5-FU [4]. Vieira de Oliveira also evaluated *MTAP* expression in two groups of breast cancer patient samples, including fresh tumors and paired normal breast tissue, as well as formalin-fixed paraffin embedded (FFPE) core breast cancer samples diagnosed as Luminal-A tumors and TNBC. Although the difference in *MTAP* expression between fresh tumors and normal tissue was not statistically significant, *MTAP* expression was significantly higher in Luminal-A breast tumors compared to TNBC. This suggests that a lack of *MTAP* expression is associated with more aggressive breast tumors and may support the development of new therapeutic approaches based on *MTAP* status in TNBC. In our study, BRCA1 mutation was more frequent in *MTAP* loss MBC (10% vs. 4%; *p* < 0.0001) which was likely associated with the increased TNBC cases.

There is growing evidence that *MTAP* can control tumor invasion and migration through many signaling mechanisms. In esophageal cancer, *MTAP* depletion can activate the GSK3/Slug/E-cadherin axis, promoting migration and invasion [14]. In colorectal cancer, downregulation of *MTAP* can also influence the epithelial-to-mesenchymal shift and stimulate tumor growth and metastasis [30]. When *MTAP* is downregulated in melanoma, 5′-methylthioadenosine (MTA) builds up and promotes tumor spread by preventing protein methylation and activating the extracellular signal-regulated kinase (ERK) signal [28]. Emerging studies show that *MTAP* overexpression dramatically changes the amounts of polyamine metabolites (particularly putrescine) in breast cancer cells [15]. These results are confirmed by the fact that re-expressing *MTAP* causes loss of anchorage-independent growth *in vitro* and loss of tumor development *in vivo* in MCF-7 breast cells that have had *MTAP* deleted [31].

MTA is a byproduct of the production of polyamines, and *MTAP* is the only metabolic enzyme that breaks it down into adenine and methylthioribose-1-phosphate (MTR-1-P) [32]. ODC, being the rate limiting enzyme in putrescine formation, is regarded as an independent predictor of a poor clinical outcome in breast cancer [24, 33–35]. ODC and polyamine metabolism have been linked to the proliferation and spread of tumor cells, according to numerous research [36–39]. Overexpression of ODC was strikingly linked with lymph node metastases, lymphovascular invasion in esophageal and breast cancers [40, 41]. *MTAP* may help prevent the growth and spread of the disease by controlling the ODC activity and putrescine level in breast cancer cells, and by limiting tumor angiogenesis by reducing ODC activity and downregulating the levels of angiogenesis mediators matrix metalloproteinase-2 (MMP2) and Vascular Endothelial Growth Factor D (VEGFD) in breast cancer cells [15].

### *MTAP* in various malignancies

Apart from the previously mentioned malignancies such as melanoma, esophageal and colorectal carcinoma, *MTAP* has a role to play in different malignancies.

Hellerband et al. [42] detected a decreased or even undetectable *MTAP* expression in three hepatocellular carcinoma lines and strong cytoplasmatic immunosignals were detectable in surrounding non-tumorous hepatocytes. These findings highlight that the downregulation or loss of *MTAP* expression in hepatocytes occurs during malignant transformation. Furthermore, Kirovski et al. [42] revealed that downregulation of *MTAP* in hepatocellular carcinoma increases MTA levels in hepatocellular carcinoma and can potentially be involved in HCC progression.

In osteosarcomas, Miyazaki et al. found that [43] *MTAP* deficiency was caused by *MTAP* gene deletion or promoter methylation in most *MTAP*-negative samples. In *in vitro* experiment, the *MTAP*-negative parental cell line was found to be more sensitive to inhibitors of *de novo* AMP synthesis, compared to the *MTAP*-positive transfectoma. The authors suggested that the *MTAP* deficiency frequently observed in osteosarcoma can be targeted with inhibitors of *de novo* purine synthesis, as a potential chemotherapy strategy for *MTAP*-negative osteosarcoma patients [43].

Zimling, Jorgensen, and Santoni-Rugiu conducted a study where they analyzed *MTAP* reactivity in 99 cases of malignant pleural mesothelioma (MPM). They found that 65% of the tumors showed decreased *MTAP* reactivity. The authors suggested that this decrease in *MTAP* expression, along with other common markers, could be a valuable diagnostic tool for MPMs. Similarly, the reduced expression of *MTAP* in triple-negative breast cancer could serve as both a diagnostic and therapeutic marker. Low *MTAP* expression has been linked to a poor prognosis in glioblastoma [44], gastric cancer [45], and non-small cell lung cancer [46], according to earlier research.

### Utilizing *MTAP* in treatment

Cytotoxic chemotherapy, radiotherapy, hormonal therapy, and immunotherapy have been shown to be effective in the treatment of breast cancer [47]. However, some breast cancer types, and especially TNBC, have no ongoing or maintenance treatment available. This might be due to the metabolic flexibility of cancer cells, which enables compensatory adaptations. It is believed that only a small number of tumor-specific metabolic vulnerabilities have been successfully targeted [48], and that many potential targeted therapies are under investigation, including therapies targeting *MTAP* deficiency [49].

The rationale behind *MTAP* targeted therapy is that adenine and methionine cannot be salvaged from endogenous MTA in *MTAP*-deficient cells. As a result, methionine deprivation and inhibitors of *de novo* purine synthesis are more toxic to *MTAP*-deficient cells than to *MTAP*-positive ones [50, 51]. The difficulty has been in developing a targeted therapy that takes advantage of *MTAP* deficiency and its resulting alterations in metabolism.

Different strategies based on *MTAP* status have been proposed that utilize inhibitors of *de novo* purine synthesis and the enzyme substrate MTA to specifically target and eliminate *MTAP*-negative cells [52–54].

According to several studies, *MTAP*-negative tumor cells are up to 20 times more susceptible to purine biosynthesis inhibitors such as MTX, 6-mercaptopurine, azaserine (a powerful inhibitor of the first step in purine biosynthesis), and L-alanosine, than *MTAP*-positive cells are [50, 55, 56]. The study by Hori et al., which transfected *MTAP* complementary DNA (cDNA) into a lung cancer cell line lacking *MTAP*, may have been the most convincing one demonstrating the link between *MTAP* deficiency and sensitivity to purine and methionine depletion. *MTAP* deficient cells proved to be more sensitive to purine synthesis inhibitors 5,10-dideazafolate, L-alanosine, and to methionine depletion. The *MTAP*-containing cell lines, but not the *MTAP*-deficient cell lines, were entirely rescued from these inhibitors and methionine restriction by adding MTA [56].

Other strategies to take advantage of *MTAP*-deficiency are also under investigation: MTA and adenine analogs such as 2,6-diaminopurine, 6-methylpurine, 2-fluoroadenine, 6-thioguanine (6-TG), and 5-FU that must undergo phosphoribosylation to transform into its toxic nucleosides are being studied to treat *MTAP*-deficient malignancies.

### Future direction

When MTA is supplied to healthy host cells, *MTAP* produces a significant amount of adenine. After that, adenine successfully competes with these co-administered drugs for phosphoribosylation by 5-phosphoribosyl-1-pyrophosphate (PRPP). For the drug to have harmful activity, it must be transformed to its toxic nucleotide. However, tumor cells lacking *MTAP* are unable to convert MTA into adenine. Because of this, PRPP levels are sufficient, and the co-administered drug can easily be transformed to its harmful nucleoside [57]. The significant difference in *MTAP* activity between tumor and host cells ensures a high level of treatment selectivity, making it a promising therapy for *MTAP* deficient malignancies in general, and *MTAP* deficient breast cancer.

*MTAP* loss is associated with ER-, HER2- and TNBC status, features a distinctive GL with potential to impact both targeted and immunotherapies and enables emerging clinical trials testing MTA2 and PRMT5 inhibitors for patients with clinically advanced breast cancer.

## MATERIALS AND METHODS

The central laboratory (Foundation Medicine, Cambridge, MA, USA) used for comprehensive genomic profiling (CGP) is Clinical Laboratory Improvement Amendments (CLIA)-certified and accredited by the College of American Pathologists. Approval for this study, including a waiver of informed consent, was obtained from the Western Institutional Review Board (Protocol No. 20152817). A minimum of 50 ng of DNA was extracted from 7,301 cases of clinically advanced ductal and lobular breast cancers. Samples used for sequencing featured a minimum of 20% tumor nuclei. After DNA extraction and DNA library preparation, adaptor-ligation based hybrid capture was performed for all coding exons from 324 cancer-related genes plus select introns from 28 genes frequently rearranged in cancer. The Illumina HiSeq instrument was used for DNA sequencing to a mean exon coverage depth of >550X [58, 59]. Tumor mutational burden (TMB) was determined using 0.9 to 1.1 Mb of sequenced DNA [60]. Microsatellite instability (MSI) status was determined on 95 loci [61]. Given that no normal DNA sample was included from each patient, a computational approach was utilized to distinguish somatic vs. germline origin of genomic alterations [62]. PD-L1 expression was determined by immunohistochemistry using 5-micron tissue sections. Following the CDx assay guidelines a tumor proportion score (TPS) was determined for each sample stained with the DAKO 22C2 CDx assay. TPS = (positive tumor cells/total tumor cell) × 100. TPS of 0% was defined as negative, low-level staining defined as 1–49% TPS, and high-level staining defined as ≥50% TPS.

Differences in sample medians were assessed using the unpaired Mann–Whitney–Wilcoxon test. Differences among categorical variables were assessed using chi square test with Yates correction. Statistical tests were 2-sided and used a significance threshold of *p* < 0.05. Reported *p* values were not adjusted for multiple testing.

## Abbreviations

5-FU: 5-fluorouracil
6-TG: 6-thioguanine
AZA: 5’aza-deoxycytidine
cDNA: Complementary DNA
HR: hormone receptor
HER2: human epidermal growth factor receptor 2
ERK: Extracellular Signal-Regulated Kinase
MMP2: Matrix Metalloproteinase-2
MTA: 5′-Methylthioadenosine
*MTAP*: Methylthioadenosine Phosphorylase
MTR-1-P: Methylthioribose-1-Phosphate
MTX: Methotrexate
ODC: Ornithine decarboxylase
PRPP: 5-phosphoribosyl-1-pyrophosphate
TNBC: Triple-Negative Breast Cancer
