# Addendum: Genomic landscape of metastatic breast cancer (MBC) patients with methylthioadenosine phosphorylase (*MTAP*) loss

**DOI:** 10.18632/oncotarget.28481

**Published:** 2023-08-07

**Authors:** Maroun Bou Zerdan, Prashanth Ashok Kumar, Elio Haroun, Nimisha Srivastava, Jeffrey Ross, Abirami Sivapiragasam

**Affiliations:** ^1^Department of Internal Medicine, SUNY Upstate Medical University, Syracuse, NY 13210, USA; ^2^Department of Internal Medicine, Division of Hematology Oncology, SUNY Upstate Medical University, Syracuse, NY 13210, USA; ^3^SUNY Upstate Medical University, Syracuse, NY 13210, USA; ^4^Foundation Medicine, Inc., Morrisville, NC 27560, USA; ^5^Departments of Pathology and Urology, SUNY Upstate Medical University, Syracuse, NY 13210, USA

**This article has an addendum:** No external funding was provided for this research.

Original article: Oncotarget. 2023; 14:178–187. 178-187. https://doi.org/10.18632/oncotarget.28376

